# Gut microbiome’s causal role in head and neck cancer: findings from mendelian randomization

**DOI:** 10.3389/fonc.2024.1453202

**Published:** 2024-11-19

**Authors:** Meng Lian, Minghong Sun, Boxuan Han, Ancha Baranova, Hongbao Cao, Fuquan Zhang

**Affiliations:** ^1^ Department of Otorhinolaryngology Head and Neck Surgery, Beijing Tongren Hospital, Capital Medical University, Beijing, China; ^2^ Key Laboratory of Otorhinolaryngology Head and Neck Surgery (Capital Medical University), Ministry of Education, Beijing, China; ^3^ Department of Otorhinolaryngology Head and Neck Surgery, Qingdao Municipal Hospital, Qingdao, China; ^4^ School of Systems Biology, George Mason University, Manassas, VA, United States; ^5^ Research Centre for Medical Genetics, Moscow, Russia; ^6^ Department of Psychiatry, The Affiliated Brain Hospital of Nanjing Medical University, Nanjing, Jiangsu, China; ^7^ Institute of Neuropsychiatry, The Affiliated Brain Hospital of Nanjing Medical University, Nanjing, Jiangsu, China

**Keywords:** gut microbiome, head and neck cancer, Mendelian randomization, enrichment analysis, functional annotation

## Abstract

**Introduction:**

The gut microbiome (GM) has been implicated in cancer pathogenesis and treatment, including head and neck cancers (HNC). However, the specific microbial compositions influencing HNC and the underlying mechanisms remain largely unknown.

**Methods:**

This study utilized published genome-wide association studies (GWAS) summary data-based two-sample Mendelian randomization (MR) to uncover the GM compositions that exert significant causal effects on HNC. Functional annotation and enrichment analysis were conducted to better understand the significant genetic variables and their connection with HNC. The HNC dataset included 2,281 cases and 314,193 controls. The GM GWAS data of 211 gut taxa (35 families, 20 orders, 16 classes, 9 phyla, and 131 genera) were obtained from the MibioGen consortium, involving 18,340 participants.

**Results:**

MR analysis revealed four GM compositions exerting causal effects on HNC. Specifically, *family Peptococcaceae.id.2024* was significantly associated with a 35% reduced risk of HNC (OR=0.65; 95%CI=0.48-0.90; *P=0.0080*). In contrast, *genus DefluviitaleaceaeUCG-011.id.11287* (OR=1.54; 95%CI=1.13-2.09; *P=0.0060*), *genus Gordonibacter.id.821* (OR=1.23; 95%CI=1.05-1.45; *P=0.012*), and *genus Methanobrevibacter.id.123* (OR=1.28; 95%CI=1.01-1.62; *P=0.040*) showed a significant association with an increased risk of HNC. These GMs interact with genes and genetic variants involved in signaling pathways, such as GTPase regulation, influencing tumor progression and disease prognosis.

**Conclusions:**

Our study demonstrates, for the first time, the causal influence of specific gut microbiome compositions on HNC, offering significant insights for advancing clinical research and personalized treatments. The identified GMs may serve as potential biomarkers or therapeutic targets, paving the way for innovative approaches in HNC diagnosis, prevention, and therapy.

## 1 Introduction

Head and neck cancer (HNC) encompasses a range of malignancies that originate in areas such as the oral cavity, throat, larynx, salivary glands, and nasal passages, posing a significant global health issue (1). This disease is notably prevalent, with millions of new cases diagnosed each year. Key risk factors, including tobacco and alcohol consumption, human papillomavirus (HPV) infection, and various environmental exposures, contribute to its widespread occurrence (2). Early detection is critical as it greatly affects the prognosis; HNC diagnosed at an early stage often responds well to treatment, resulting in high survival rates. Conversely, advanced-stage HNC presents more treatment challenges and has a less favorable prognosis (3). Efforts in raising awareness and promoting prevention are essential to reduce the disease’s impact (4).

The human microbiome is a dynamic community that colonizes various organs, with the gut being particularly abundant in microbial species due to its unique structure and role (5). The gut microbiome (GM) includes around 1500 distinct species (6), predominantly belonging to four major bacterial phyla: *Firmicutes, Bacteroidetes, Proteobacteria*, and *Actinobacteria* (7). These bacteria are involved in a multitude of immune and metabolic activities in the gut. Studies reveal that the gut microbiome and its metabolic products have a substantial impact on the host’s physiology, influencing processes such as vitamin synthesis, production of intestinal hormones, maintenance of intestinal barrier integrity, and the digestion and absorption of nutrients (8).

Cancer can disrupt the balance of the GM, affecting immune modulation and metabolite production, which in turn impacts the prognosis and treatment outcomes of various cancer types. Understanding and manipulating the GM may provide novel therapeutic avenues for cancer prevention and treatment, highlighting the importance of considering the microbiome in cancer research and patient care (9–12). Specific microbial compositions and metabolites have been linked to different cancer types including HNC (13), further emphasizing the intricate relationship between the GM and cancer pathogenesis and treatment (14, 15). Exploring the intricate interactions between the gut microbiome and HNC could unveil novel avenues for HNC prevention, early detection, and therapeutic interventions.

Few studies have examined the link between the GM and HNC, highlighting the importance of understanding the connection between the intestinal microbiome and cancer treatments in HNCs (13). Notably, no prior research has investigated the causal effect of GM on HNC at the genetic level. Our hypothesis is that certain GM compositions, if not all, exert a causal effect on the risk of HNC. Identifying these GMs could offer significant insights into their role in cancer progression, which would not only enhance clinical research but also pave the way for more targeted and personalized treatment approaches for HNC.

Mendelian randomization (MR) analysis is a method that evaluates the causal relationship between risk factors and outcomes by using genetic variants associated with the risk factors as instrumental variables (IVs) (16). This approach can provide stronger genetic evidence and enhance the validity of causal inferences. In this study, we utilized the most comprehensive and recent GWAS summary data on human GM to deeply explore the genetic relationships involved.

## 2 Methods

**Figure 1:**
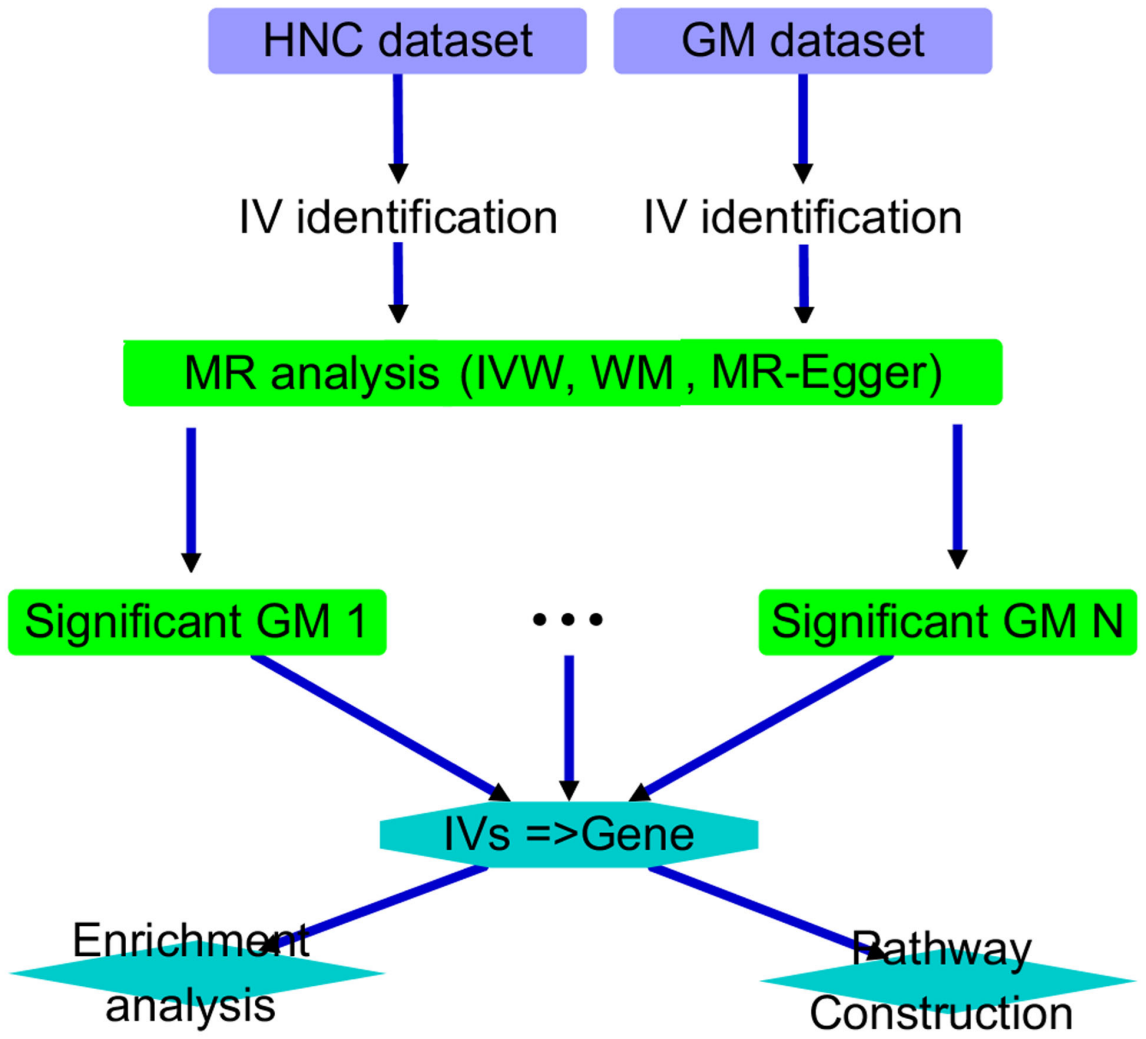
Overall workflow diagram of the study.

This study was structured as follows. First, we conducted an MR analysis on each of the 211 GM datasets to investigate the causal relationship between the corresponding GM taxa and HNC. We identified GM taxa with significant causal relationships for further analysis and discussion. Specifically, an AI-based literature data mining was performed to explore the connection between their IVs and corresponding genes and HNC. Functional pathways were then constructed accordingly. Please see **Figure 1** for the overall workflow of the study.

### 2.1 Data source

The GWAS summary data utilized in this study were all obtained from publicly available datasets. Inclusion Criteria for HNC Datasets: The data for both HNC cases and controls were obtained from publicly available datasets, specifically the FinnGen R10 database (https://r10.risteys.finngen.fi/) (17). The HNC group comprised 2,281 cases identified using International Classification of Diseases (ICD) codes (phenocode: C3_HEAD_AND_NECK_EXALLC), while the control group included 314,193 individuals without HNC. Due to the reliance on summary-level genetic data from genome-wide association studies (GWAS), detailed clinical and demographic information—such as age, gender, comorbidities, and lifestyle factors—were not available. This limitation is further emphasized by the absence of granular patient-level data. However, the dataset predominantly includes participants of European ancestry, which ensures consistency in genetic background across both cases and controls. The alleles and effects of the two datasets were harmonized before the analysis. The GM GWAS data of 211 gut taxa (35 families, 20 orders, 16 classes, 9 phyla, and 131 genera) were obtained from the MibioGen consortium, involving 18,340 participants (18). Fifteen gut taxa without known names were excluded from the analysis.

### 2.2 MR analysis

The primary Mendelian randomization (MR) analysis utilized the inverse-variance weighted (IVW) approach, supplemented by the weighted median and MR-Egger methods, as implemented in TwoSampleMR (19). The primary MR analysis aimed to infer causal relationships between gut microbiome compositions and HNC. We utilized several statistical methods as implemented in TwoSampleMR (19): Inverse-Variance Weighted (IVW): This approach combines estimates from multiple single-nucleotide polymorphisms (SNPs) while accounting for their variances, providing a robust estimate of causal effects. Weighted Median: This method estimates causal effects by giving more weight to SNPs with more accurate estimates, allowing for valid causal inference even if some SNPs are invalid instruments. MR-Egger: This method estimates causal effects while allowing for directional pleiotropy. The intercept from the MR-Egger model can indicate the presence of pleiotropy, helping to assess the validity of the instrumental variables used.

For the MR analysis, single nucleotide polymorphisms (SNPs) were chosen as instrumental variables (IVs) based on their strong association with GM taxa from threshold of *P < 1×10^–5^*, ensuring that only SNPs with robust associations were included as IVs. To avoid linkage disequilibrium, we applied a clumping threshold of r² < 0.001 within a 10 Mb window, ensuring that the selected SNPs were independent.

To address potential biases due to insufficient IVs, we employed multiple MR methods, including the Inverse-Variance Weighted (IVW) approach, which provides the primary estimate of causal effects. Additionally, Weighted Median and MR-Egger methods were used to account for potential biases such as pleiotropy (where SNPs influence multiple traits). These additional methods offer robust estimates even if some IVs are invalid, helping mitigate the risk of bias from weak or insufficient instruments. Heterogeneity tests (e.g., Cochran’s Q and I² statistics) were also conducted to assess the consistency of the results across the SNPs, with thresholds of *P < 0.05* and I^2^ > 0.25, respectively (20). This study adhered to the STROBE-MR checklist for strengthening the reporting of observational studies utilizing Mendelian Randomization (21).

### 2.3 Functional enrichment analysis

To gain a deeper understanding of the use of instrumental variables (SNPs) in Mendelian randomization (MR) analysis connecting gut microbiota (GM) and head and neck cancers (HNCs), we performed a functional enrichment analysis on the genes mapped to these IVs using the DAVID platform (https://david.ncifcrf.gov/). The input for this analysis comprised genes corresponding to IVs derived from GM data used in MR analysis. The IVs were mapped to genes using Entrez.elink (https://www.ncbi.nlm.nih.gov/books/NBK25500/#_E-utilities_5._eLink). This analysis involved examining Gene Ontology (GO) terms and Kyoto Encyclopedia of Genes and Genomes (KEGG) pathways. The main goal was to explore the molecular functions, biological processes, and cellular components associated with the target genes and their gene products. Additionally, it aimed to investigate their roles in various biological pathways, including those related to metabolism, signaling, and diseases.

### 2.4 Knowledge-based pathway construction

To better understand the use of instrumental variables (SNPs) in MR analysis linking GM and HNCs, we conducted a thorough data mining analysis using an AI-powered tool from AIC LLC (https://www.gousinfo.com/en/advancedsearch.html). This process involved creating molecular pathways connecting GM and HNCs via genes linked to the chosen SNPs. We hypothesized that these SNPs influence molecular pathways bridging GM and HNCs. To validate this, we reviewed relevant references and statements about the target genes, ensuring quality by excluding unrelated associations. We then constructed molecular pathways connecting GM and HNC based on the identified correlations.

## 3 Results

### 3.1 MR analysis result

In the MR analysis, five datasets were excluded due to insufficient instrumental variables (IVs). Our results indicate that *genus DefluviitaleaceaeUCG011.id.11287, genus Gordonibacter.id.821*, and *genus Methanobrevibacter.id.123* were linked to an increased disease risk, whereas family *Peptococcaceae.id.2024* was associated with a reduced disease risk. The results are presented in **Table 1**. Here, *Methanobrevibacter* spp. is a genus of archaea within the Kingdom *Euryarchaeota* and family *Methanobacteriaceae*,. Archaea are distinct from bacteria and eukaryotes, possessing unique metabolic pathways, which may contribute to their role in disease pathogenesis.

**Table 1:** MR analysis results of four GM taxa on HNC.

Exposure	Outcome	Method	#IV	P_IV	b (se)	OR [95%CI]	P
GM 2024	HNC	IVW	9	1.00E-05	-0.427 (0.162)	0.65 [0.48-0.90]	8.40E-03
GM 2024	HNC	WM	9	1.00E-05	-0.097 (0.218)	0.91 [0.59-1.39]	0.66
GM 2024	HNC	MR-Egger	9	1.00E-05	-0.217 (0.470)	0.80 [0.32-2.02]	0.66
GM 11287	HNC	IVW	9	1.00E-05	0.430 (0.156)	1.54 [1.13-2.09]	5.76E-03
GM 11287	HNC	WM	9	1.00E-05	0.319 (0.208)	1.38 [0.92-2.07]	0.13
GM 11287	HNC	MR-Egger	9	1.00E-05	0.786 (0.583)	2.19 [0.70-6.88]	0.22
GM 821	HNC	IVW	11	1.00E-05	0.210 (0.084)	1.23 [1.05-1.45]	0.012
GM 821	HNC	WM	11	1.00E-05	0.159 (0.112)	1.17 [0.94-1.46]	0.16
GM 821	HNC	MR-Egger	11	1.00E-05	0.638 (0.355)	1.89 [0.94-3.80]	0.11
GM 123	HNC	IVW	6	1.00E-05	0.247 (0.120)	1.28 [1.01-1.62]	0.040
GM 123	HNC	WM	6	1.00E-05	0.321 (0.153)	1.38 [1.02-1.86]	0.036
GM 123	HNC	MR-Egger	6	1.00E-05	0.355 (0.447)	1.43 [0.59-3.42]	0.47

HNC, Head and neck cancer; GM 2024: family.Peptococcaceae.id.2024; GM 11287: genus.DefluviitaleaceaeUCG011.id.11287; GM 821: genus.Gordonibacter.id.821; GM 123: genus.Methanobrevibacter.id.123.

**Figure 2:**
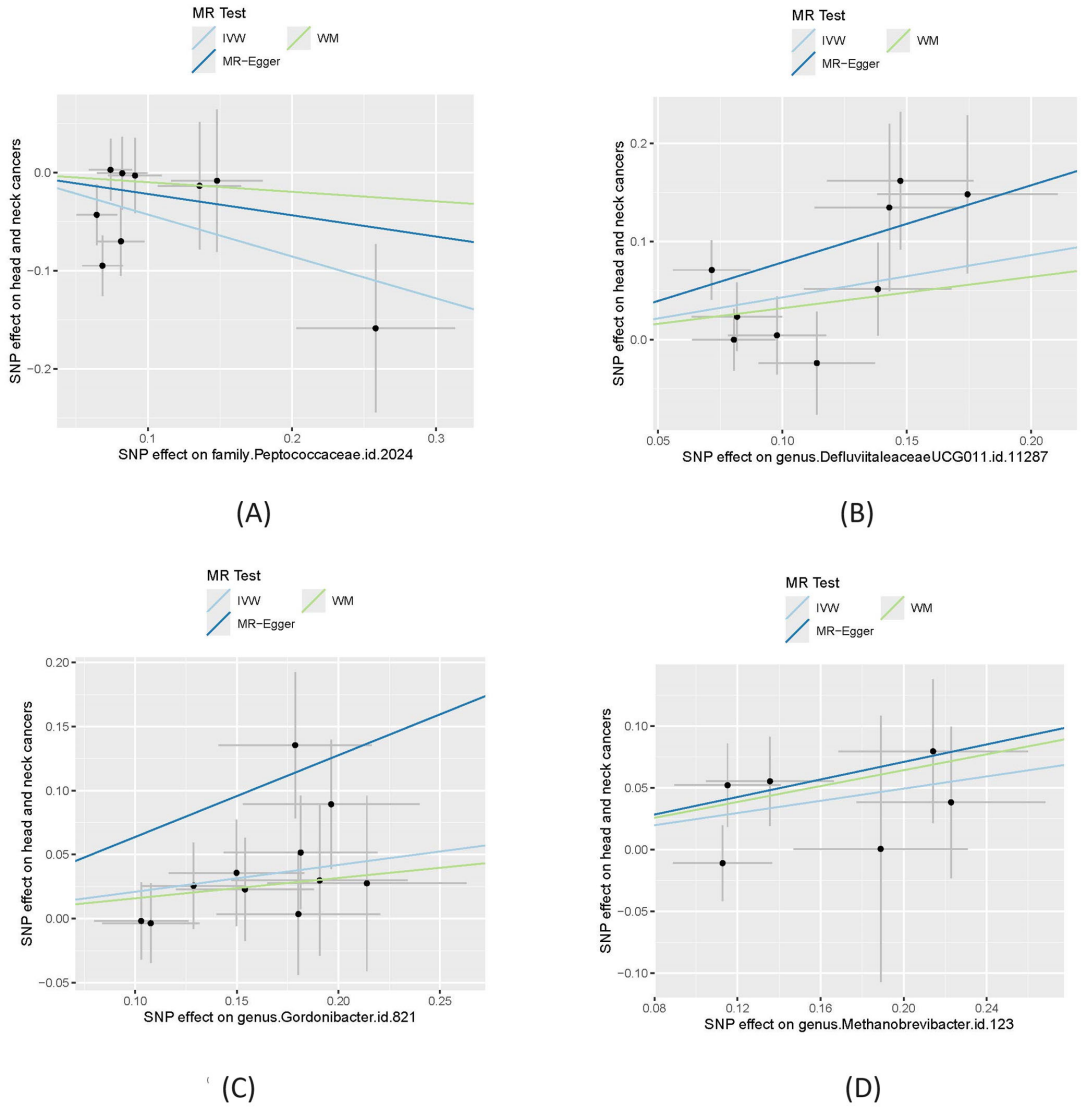
Causal effect of four gut microbiomes on head and neck cancers. **(A)** Effect of *family.Peptococcaceae.id.2024*; **(B)** effect of *genus.DefluviitaleaceaeUCG011.id.11287*; **(C)** Effect of *genus.Gordonibacter.id.821*; **(D)** Effect of *genus.Methanobrevibacter.id.123*. In each plot, the lines were the effect sizes **(B)** of the MR analysis. IVW, inverse variance weighted; WM, weighted median.

**Table 1** shows that, using the IVW method, the analysis revealed that *family.Peptococcaceae.id.2024* was significantly associated with a reduced risk of HNC. The odds ratio (OR) =0.65 with a 95% confidence interval (CI) = 0.48-0.90, and the p-value was 0.0084, indicating a statistically significant protective effect. The WM and MR-Egger methods, however, did not show significant associations. The WM method yielded an OR=0.91 (95% CI= 0.59-1.39) with a p-value of 0.655, while the MR-Egger method produced an OR=0.80 (95% CI=0.32-2.02) with a *P=0.66*. These methods may not have shown significance due to their lower statistical power compared to the IVW method, especially when the effect size is modest. We also visualize these results in scatter plot as shown in **Figure 2A**.

Additionally, heterogeneity analysis suggested that the directions of causal effects were consistent across the different techniques. No directional pleiotropy (*P > 0.05* and MR-Egger intercept < 0.01) or heterogeneity (*P > 0.05*) was detected, indicating that the study results are reliable and the lack of significance in the WM and MR-Egger methods does not undermine the overall findings.

**Figure 3:**
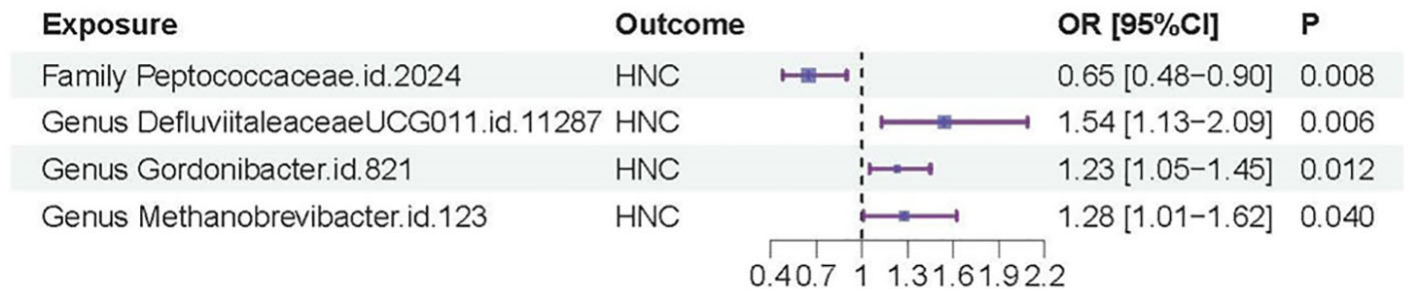
Forest plot of the casual effects of the four GMs on HNC.

In contrast, *genus.DefluviitaleaceaeUCG011.id.11287* (OR=1.54; 95%CI=1.13-2.09; *P= 0.0058*), *genus.Gordonibacter.id.821* (OR=1.23; 95%CI=1.05-1.45; *P = 0.012*), and *genus.Methanobrevibacter.id.123* (OR=1.28; 95%CI=1.01-1.62; *P=0.040*) showed a significant association with an increased risk of HNC, as shown in **Figures 2B–D**. Similar to *family.Peptococcaceae.id.2024*, the WM and MR-Egger methods did not show significant associations for most tests due to their lower statistical power compared to the IVW method. However, no directional pleiotropy (*P > 0.05*, MR-Egger intercept < 0.01) or heterogeneity (*P > 0.05*) was detected, indicating that the study results are reliable. The lack of significance in the WM and MR-Egger methods does not undermine the overall findings. We present a forest plot illustrating the causal effects of the four GMs on HNC, as determined by the main method (IVW), in **Figure 3**.

**Figure 4:**
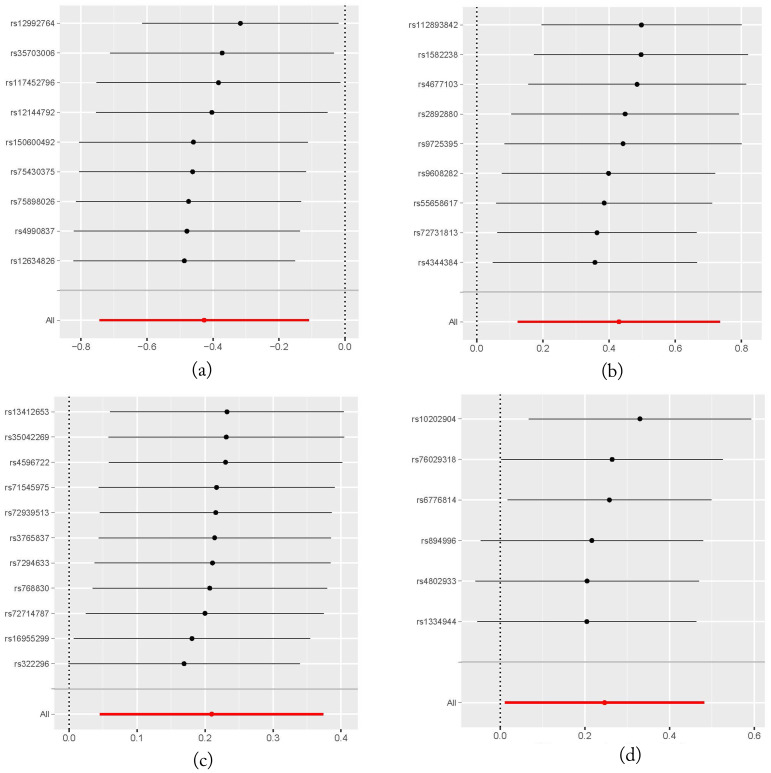
Leave-One-Out plots illustrating the robustness of the MR analysis for four gut microbiota taxa. **(A)** family.Peptococcaceae.id.2024 on head and neck cancers; **(B)** genus.DefluviitaleaceaeUCG011.id.11287 on head and neck cancers; **(C)** genus.Gordonibacter.id.821 on head and neck cancers; **(D)** genus.Methanobrevibacter.id.123 on head and neck cancers.

It should be noted that none of the four taxa showed significance after FDR correction (*P > 0.05*), likely due to the small number of IVs used in our MR analysis, which resulted in relatively larger p-values. Moreover, Leave-One-Out (LOO) sensitivity analysis showed that the results for one of the four gut microbiota (genus.Methanobrevibacter.id.123) were influenced by removing one instrumental variable (rs10202904). This may be due to the fact that only six IVs were used in this analysis. However, for the other three gut microbiota, removing any single IV did not significantly influence the results, suggesting the robustness of the MR analysis outcomes. The LOO plot is presented in **Figure 4**.

### 3.2 Analysis of IVs with enrichment analysis

**Table 2** lists the IVs with mapped genes for each of the four GM presented in **Table 1**. Please note, here we only listed the ones with at least one mapped genes, as enrichment analysis only take gene symbols as inputs.

**Table 2:** IVs with mapped genes of four GM presenting significant casual effect on HNC.

GM	IVs(SNPs)	Mapped genes
*genus.Methanobrevibacter.id.123*	rs10202904	CNTNAP5
*genus.Methanobrevibacter.id.123*	rs1334944	DUSP5
*genus.Methanobrevibacter.id.123*	rs4779844	LINC02352
*genus.Methanobrevibacter.id.123*	rs4779844	LINC03034
*genus.Methanobrevibacter.id.123*	rs6776814	NR2C2
*genus.Gordonibacter.id.821*	rs13412653	LOC101929418
*genus.Gordonibacter.id.821*	rs322296	PTN
*genus.Gordonibacter.id.821*	rs35042269	ABLIM2
*genus.Gordonibacter.id.821*	rs3765837	HHAT
*genus.Gordonibacter.id.821*	rs76287110	LOC100131635
*genus.Gordonibacter.id.821*	rs76287110	BCL6
*genus.Gordonibacter.id.821*	rs768830	HDAC9
*genus.DefluviitaleaceaeUCG011.id.11287*	rs112893842	PTPRD
*genus.DefluviitaleaceaeUCG011.id.11287*	rs1582238	SPAG17
*genus.DefluviitaleaceaeUCG011.id.11287*	rs4344384	LOC105378334
*genus.DefluviitaleaceaeUCG011.id.11287*	rs4677103	LINC00870
*genus.DefluviitaleaceaeUCG011.id.11287*	rs55658617	DSCAM
*genus.DefluviitaleaceaeUCG011.id.11287*	rs72731813	SLC10A7
*genus.DefluviitaleaceaeUCG011.id.11287*	rs9608282	ADORA2A
*genus.DefluviitaleaceaeUCG011.id.11287*	rs9608282	SPECC1L
*family.Peptococcaceae.id.2024*	rs117452796	PTPRD
*family.Peptococcaceae.id.2024*	rs12634826	B3GNT5
*family.Peptococcaceae.id.2024*	rs12634826	MCF2L2
*family.Peptococcaceae.id.2024*	rs150600492	LOC105378551
*family.Peptococcaceae.id.2024*	rs150600492	DOCK1
*family.Peptococcaceae.id.2024*	rs4990837	CSMD1
*family.Peptococcaceae.id.2024*	rs75430375	LOC102724637
*family.Peptococcaceae.id.2024*	rs75898026	MCF2L

Enrichment analysis revealed that only the genes corresponding to IVs from *family.Peptococcaceae.id.2024* were significantly enriched in two pathways: Guanine-nucleotide releasing factor (GNRF) and Guanyl-nucleotide exchange factor (GEF) activity. These pathways are essential for regulating small GTPases, which are crucial molecules in cell growth and migration. In contrast, genes corresponding to IVs from the other three gut microbiota did not show significant enrichment in any pathways, indicating that their genes were not functionally collaborating on specific functions.

### 3.3 Knowledge-based pathway connecting HNC and four GMs

**Figure 5:**
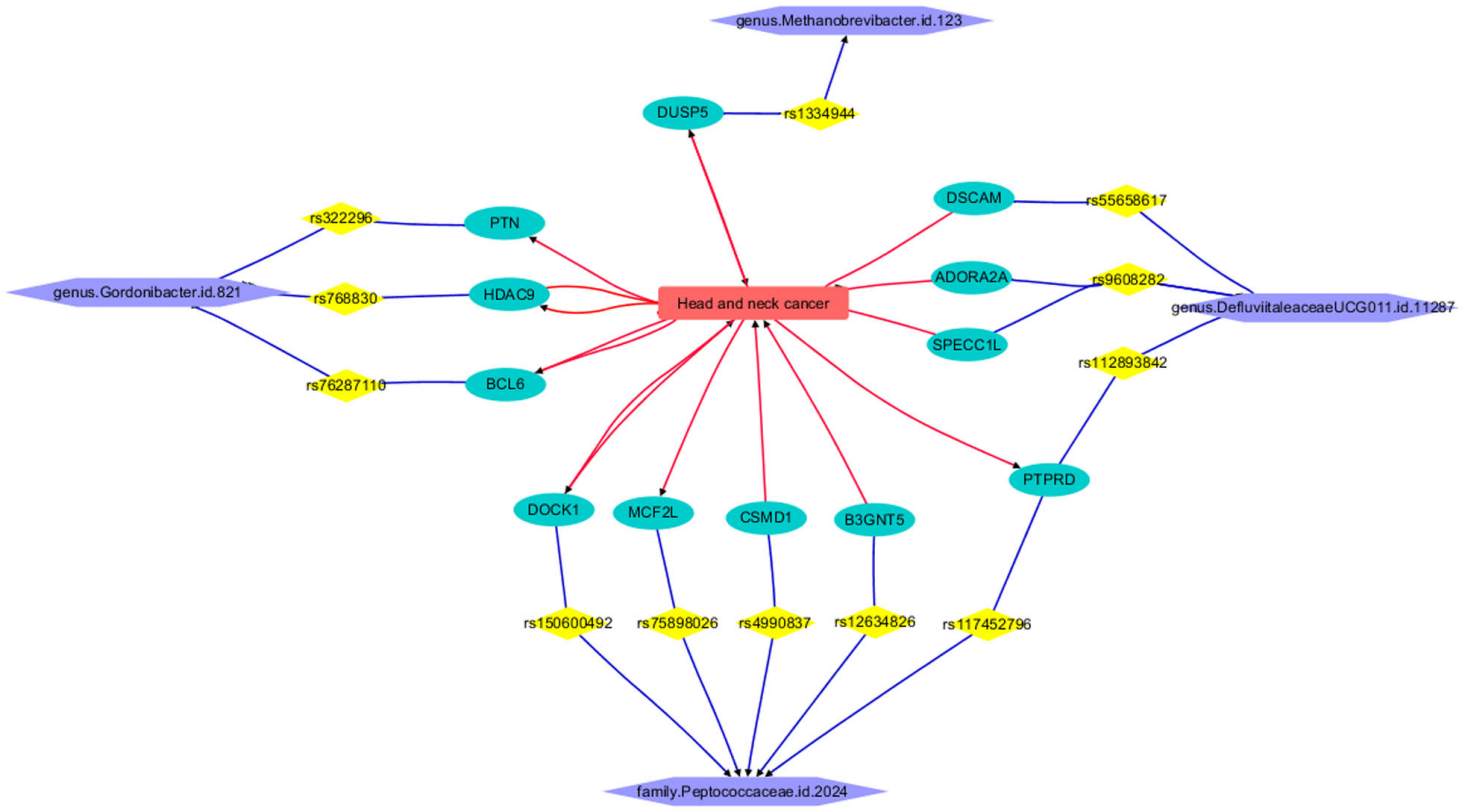
Functional pathway connecting HNC and four GMs.

The LDM process revealed that HNC is connected to 12 out of the 27 genes corresponding to GM IVs, forming a functional pathway that links HNC with the four GMs, as illustrated in **Figure 5**. Each relationship or edge between HNC and the genes was supported by one or more references. The constructed pathway helps depict the mechanism by which genetic liability to the four types of GMs influences the pathological development and progression of HNC. It is important to note that none of the IVs (SNPs) have been reported to have a direct relationship with HNC, nor do the remaining 15 out of the 27 genes corresponding to GM-related IVs. This finding underscores the need for further research on these genes and genetic variants.

**Figure 6:**
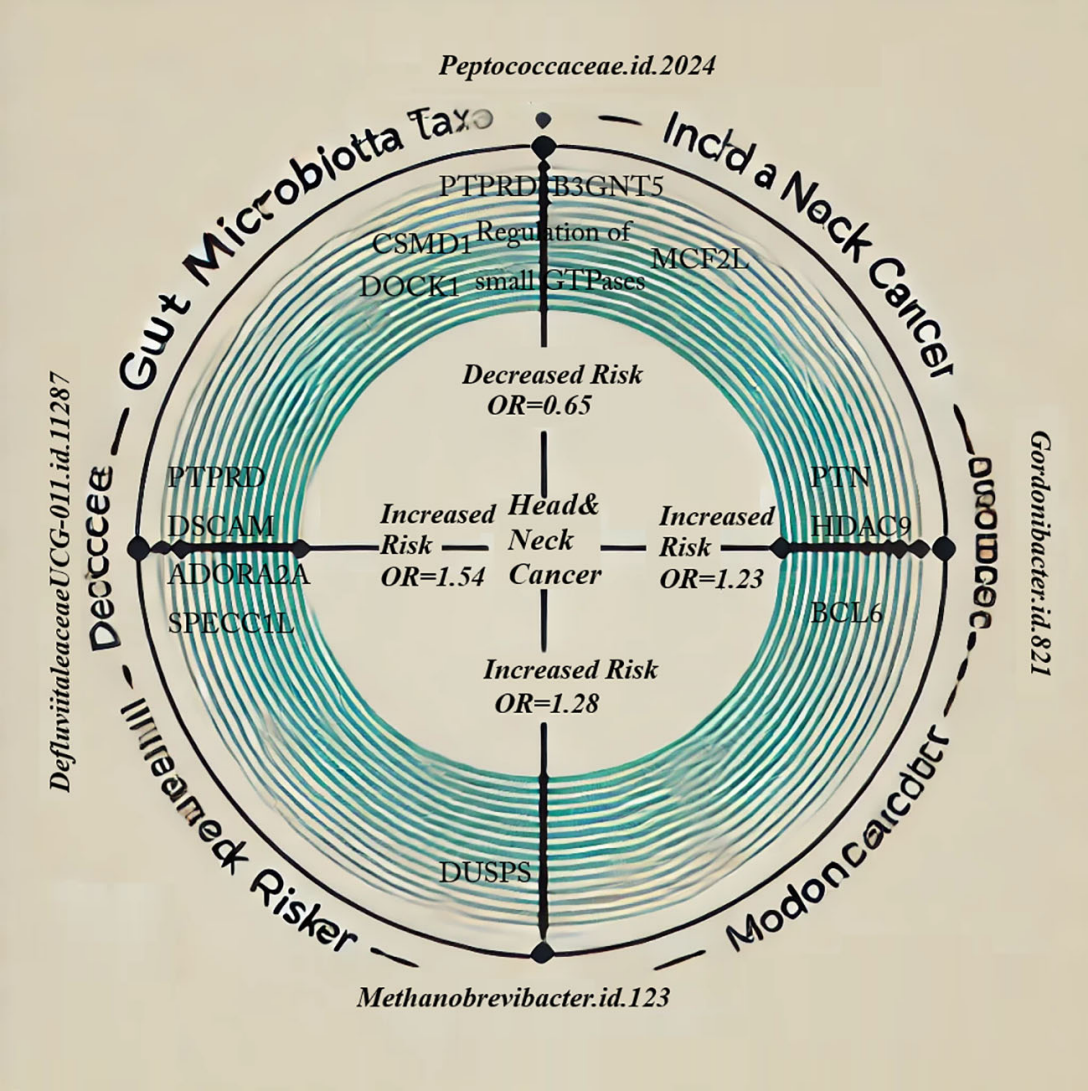
Ring diagram showing the causal association between four gut microbiota and head and neck cancer: *Family Peptococcaceae* (reduced risk)*; Genus Defluviitaleaceae UCG-011* (increased risk)*; Genus Gordonibacter* (increased risk)*; Genus Methanobrevibacter* (increased risk).

We have integrated the findings from our MR analysis, enrichment analysis, and pathway analysis to create the Ring diagram (Circus plot), illustrating the association between the four GM taxa and HNC, as shown in **Figure 6**.

## 4 Discussion

The gut microbiome (GM) has been implicated in cancer pathogenesis and treatment, including head and neck cancers (HNC) (13). However, the specific microbial compositions influencing HNC and the underlying mechanisms remain largely unknown. In this study, we used Mendelian randomization (MR) analysis to explore the causal effect of 211 GM compositions on HNC, identifying four with significant causal effects: *family Peptococcaceae (id.2024), genus Defluviitaleaceae UCG-011 (id.11287), genus Gordonibacter (id.821)*, and *genus Methanobrevibacter (id.123)*. Further analysis of the instrumental variables (SNPs) and the corresponding genes revealed functional pathways and networks linking HNC to these four GMs. These findings enhance our understanding of the relationships between the gut microbiome and HNC.

Our MR analysis identified that genus *DefluviitaleaceaeUCG011.id.11287*, *genus Gordonibacter.id.821*, and *genus Methanobrevibacter.id.123* were associated with an increased risk of disease, whereas *family Peptococcaceae.id.2024* was linked to a decreased risk of disease (**Table 1**). Despite the WM and MR-Egger methods not showing significance, there was no evidence of directional pleiotropy (*P > 0.05* and MR-Egger intercept < 0.01) or heterogeneity (*P > 0.05*), suggesting that the study results are robust. The absence of significance in the WM and MR-Egger methods does not detract from the overall findings.

Enrichment analysis revealed that three genes (MCF2L, DOCK1, and MCF2L2) corresponding to the IVs from the *family Peptococcaceae.id.2024* were significantly enriched in Guanine-nucleotide releasing factor (GNRF) and Guanyl-nucleotide exchange factor (GEF) activity-related pathways (**Table 3**). These pathways are crucial for regulating small GTPases, which are pivotal molecules in cell growth and migration. GTPases play a significant role in modulating gut microbiota composition and function, with dysregulation potentially leading to health implications. This interplay between GTPases and the GM highlights their importance as potential therapeutic targets (22, 23). Additionally, the GM reciprocally impacts GTPases by modulating their activity and expression, thereby influencing immune responses, metabolism, and signaling pathways bidirectionally to maintain gut homeostasis and health (24, 25).

**Table 3:** Enrichment analysis for the genes corresponding to IVs from *family.Peptococcaceae.id.2024*.

Category	Term	Count	Overlap (%)	P-value	Genes	FDR
KW-0344	Guanine-nucleotide releasing factor	3	37.5	0.0011	MCF2L, DOCK1, MCF2L2	0.0091
GO:0005085	guanyl-nucleotide exchange factor activity	3	37.5	0.0015	MCF2L, DOCK1, MCF2L2	0.02

In HNC, dysregulation of GNRF, particularly Vav2, can lead to the abnormal activation of GTPases, promoting tumor progression through altered signaling pathways, ribosome biogenesis, invasion, immunosuppression, and metastasis (26–29). GNRF’s role in accelerating HNC is further supported by its enhancement of cellular activities and impact on signaling pathways, with potential therapeutic targets identified as DOCK2 and ARHGEF2 (30, 31). Additionally, studies have shown that the GEF activity of proteins such as PLEKHG4, Tiam1, and VAV2 influences cell behavior and therapeutic targets in HNC (32–34). These findings suggest that GTPase regulation is a common influential mechanism in both HNC and the *family Peptococcaceae.id.2024*.

The functional pathway illustrated in **Figure 5** demonstrates the connection of HNC to the *genus Gordonibacter.id.821* through three genes: PTN, BCL6, and HDAC9. Elevated levels of Pleiotrophin (PTN) in HNC contribute to aggressive tumor behavior and correlate with poor prognosis (35). The overexpression of BCL6 in HNC fosters tumor growth and metastasis, with BCL6 expression and neutrophil infiltration serving as significant prognostic indicators in HPV-related oropharyngeal cancer (36). Furthermore, the heightened expression of HDAC9 in HNC drives tumor progression, metastasis, and resistance to cisplatin therapy, highlighting its potential as a therapeutic target across various HNC subtypes (37–39).

The gene connecting HNC and *genus Methanobrevibacter.id.123* is DUSP5. DUSP5 plays a significant role in HNC progression. Downregulation of DUSP5 promotes tumor growth by dysregulating MAPK signaling pathways, leading to cetuximab resistance and potential influence by lncRNA-ENST00000412010 (40, 41). Conversely, upregulation of DUSP5 suppresses tumor growth, inhibiting proliferation and invasion, indicating its potential as a therapeutic target (42, 43).

Four genes, namely DSCAM, ADORA2A, SPECC1L, and PTPRD, establish a connection between the *genus DefluviitaleaceaeUCG011.id.11287* and HNC. Overexpression of DSCAM and ADORA2A in HNC has been correlated with tumor progression and a poorer prognosis, thereby identifying them as potential therapeutic targets (44, 45). SPECC1L facilitates HNC progression by modulating cell pathways. Moreover, the presence of the BCL6-SPECC1L fusion gene in nasopharyngeal carcinoma implies its relevance in this specific subtype (46). Alterations in the PTPRD gene affect tumor suppression and cell growth in HNC, suggesting its involvement in disease progression and its potential therapeutic significance (47).

It’s important to note that some IVs (SNPs) mapped to genes do not have a documented connection with HNC, nor do the IVs themselves. Additionally, most genes corresponding to these IVs were not simultaneously enriched in the same pathways, indicating their diverse functionality. This highlights the need for further research into these genes and genetic variants to elucidate their potential role in HNC.

This study has several limitations. First, all participants were of European origin, which limits the generalizability of our findings to other populations. Second, the relatively small number of HNC cases compared to controls may have reduced the statistical power to detect subtle associations. Third, the reported results regarding the microbiota are based on extremely broad taxonomic levels (family, genus, and phylum), which may result in findings that are too general and lack specificity. The use of 16S rRNA gene sequencing in the MiBioGen consortium’s GWAS data allows for detection only at these broad levels, with no genetic data available at the species level. Additionally, the negative results may carry biases related to sample size and power, warranting cautious interpretation. Furthermore, the analysis may be influenced by contamination from environmental factors and other confounding variables. Our findings are also impacted by the presence of numerous genetic variants with relatively small effect sizes, which can complicate causal inference. Moreover, none of the taxa showed significance after FDR correction, likely due to the small number of instrumental variables (IVs) used in our MR analysis, which limited statistical power and led to relatively larger p-values. Lastly, the potential for a high rate of false positives among the identified variants further underscores the need for careful interpretation of our results.

## 5 Conclusion

Our study identified four gut microbiota (GMs) with significant causal relationships with HNC at the genetic level. Specifically, genetic predisposition to the *family Peptococcaceae.id.2024* was linked to a decreased risk of HNC, while genetic predispositions to the *genus DefluviitaleaceaeUCG011.id.11287, genus Gordonibacter.id.821*, and *genus Methanobrevibacter.id.123* were associated with an increased risk of HNC. These GMs interact with genes and genetic variants involved in signaling pathways, such as GTPase regulation, influencing tumor progression and disease prognosis. Our results indicate a causal effect of certain GM microbial compositions on HNC, providing significant insights for advancing clinical research and tailoring treatment approaches for HNC.

## Data Availability

Publicly available datasets were analyzed in this study. This data can be found here: https://r10.risteys.finngen.fi/.
